# Complex relationship between growth hormone and sleep in children: insights, discrepancies, and implications

**DOI:** 10.3389/fendo.2023.1332114

**Published:** 2024-01-24

**Authors:** Marco Zaffanello, Angelo Pietrobelli, Paolo Cavarzere, Alessandra Guzzo, Franco Antoniazzi

**Affiliations:** ^1^ Department of Surgery, Dentistry, Paediatrics and Gynaecology, University of Verona, Verona, Italy; ^2^ Department of Pathology and Diagnostics, School of Medicine, University of Verona, Verona, Italy

**Keywords:** children, growth hormone, recombinant growth hormone, pediatric, sleep quality

## Abstract

Growth hormone (GH) is crucial to growth and development. GH secretion is regulated by a complex feedback system involving the pituitary gland, hypothalamus, and other organs, and predominantly occurs during deep sleep. Isolated and idiopathic growth hormone deficiency (GHD) is a condition characterized by GHD without any other signs or symptoms associated with a specific syndrome or disease. The aim of this narrative review was to evaluate the relationship between GH and sleep in children using published data. Various databases (Medline/PubMed, Scopus, and Web of Science) were systematically searched for relevant English language articles published up to April 2023. Search strategies included the terms ‘children/pediatric’, ‘growth hormone’, ‘growth hormone deficiency’ and ‘sleep’. Data were extracted by two independent reviewers; 185 papers were identified of which 58 were duplicates and 118 were excluded (unrelated n=83, syndromic/genetic GHD n=17, non-English n=13, abstract n=1, case report n=1). Overall, nine studies (six clinical studies, two case series, and one survey) were included. GHD appears to have an adverse effect on sleep in children, and GH therapy has only been shown to have a beneficial effect on sleep parameters in some individuals. Notably, identified data were limited, old/poor quality, and heterogenous/inconsistent. Further research of GHD in pediatric populations is necessary to improve the understanding of GHD impact on sleep and its underlying mechanisms, and to determine the specific impacts of GH therapy on sleep in children.

## 1 Introduction

Sleep is a vital physiological process for physical and mental well-being and is characterized by decreased brain activity and relaxed muscle tone. Sleep is regulated by intricate biological mechanisms involving the circadian rhythm, sleep neurons, and neurochemical regulation. The circadian rhythm governs the sleep-wake cycle, sleep neurons control the transition between wakefulness and sleep, and neurochemical regulation involves various nervous systems and chemical substances (1).

Sleep is fundamental in regulating the hypothalamic-pituitary-adrenal axis, which is involved in growth hormone (GH) release. During deep (slow-wave) sleep, there is an increase in GH secretion. This peak of GH during sleep is essential for growth and muscle development, as well as tissue regeneration and repair. GH has a strong association with brain activity during deep sleep and plays a role in maintaining tissue homeostasis (2, 3). Furthermore, GH has been identified as being one of the potential mechanisms that link sleep to body composition (4).

Insufficient or poor quality of sleep can have a significant impact on the development, learning, behavior, and overall health of children (5, 6) including a detrimental effect on their physical and mental well-being (7). Other effects include difficulties with concentration, increased irritability, growth impairment, weakened immune function, anxiety, and depression (7).

GH deficiency can also occur in genetic disorders such as Prader-Willi syndrome (PWS), where GH deficiency is just one of many symptoms. However, the effects of recombinant human GH (hGH) therapy on sleep disordered breathing (SDB) in children with PWS remain subject to debate, and study results vary. Significant differences in normalized body mass index have been observed between groups treated and not treated with GH, but no significant differences have been found in parameters related to obstructive sleep apnea (OSA) in these groups (8). However, it is important to note that long-term GH therapy may contribute to hypertrophy of the tonsils and adenoids, worsening nighttime apnea in children with PWS (9). Therefore, the worsening of OSA severity observed in 13% of children with PWS treated with GH supports the current recommendation to perform polysomnography after the initiation of GH therapy (10). Although GH therapy may improve sleep quality in some patients, it can also cause or worsen SDB in others, resulting in reduced overall sleep quality and quality of life (9).

Isolated and idiopathic growth hormone deficiency (GHD) occurs when there is a deficiency of GH without any specific syndrome or disease (i.e., the deficit is unrelated to a genetic disorder or a particular pathological condition). Isolated GH deficiencies can be categorized based on the age at which they manifest. Congenital GHD is present at birth (or shortly after) and is usually caused by genetic abnormalities or malformations in the hypothalamus or pituitary gland. Acquired GHD occurs due to injuries or damage to the hypothalamus or pituitary gland (e.g., via head trauma, infections, tumors, or treatments such as radiation therapy). Idiopathic GHD is when the cause of isolated GHD remains unknown. Diagnosis can be made through hormonal and imaging tests, and treatment typically involves subcutaneous injections of synthetic GH replacement therapy to stimulate growth (2).

Sleep is an important period of increased GH release (11). Indeed, a significant release of GH can be observed in conjunction with the first episode of slow-wave activity, especially shortly after sleep onset (12, 13). This indicates that slow-wave sleep appears to play an important role in the regulation of GH secretion.

**Figure 1:**
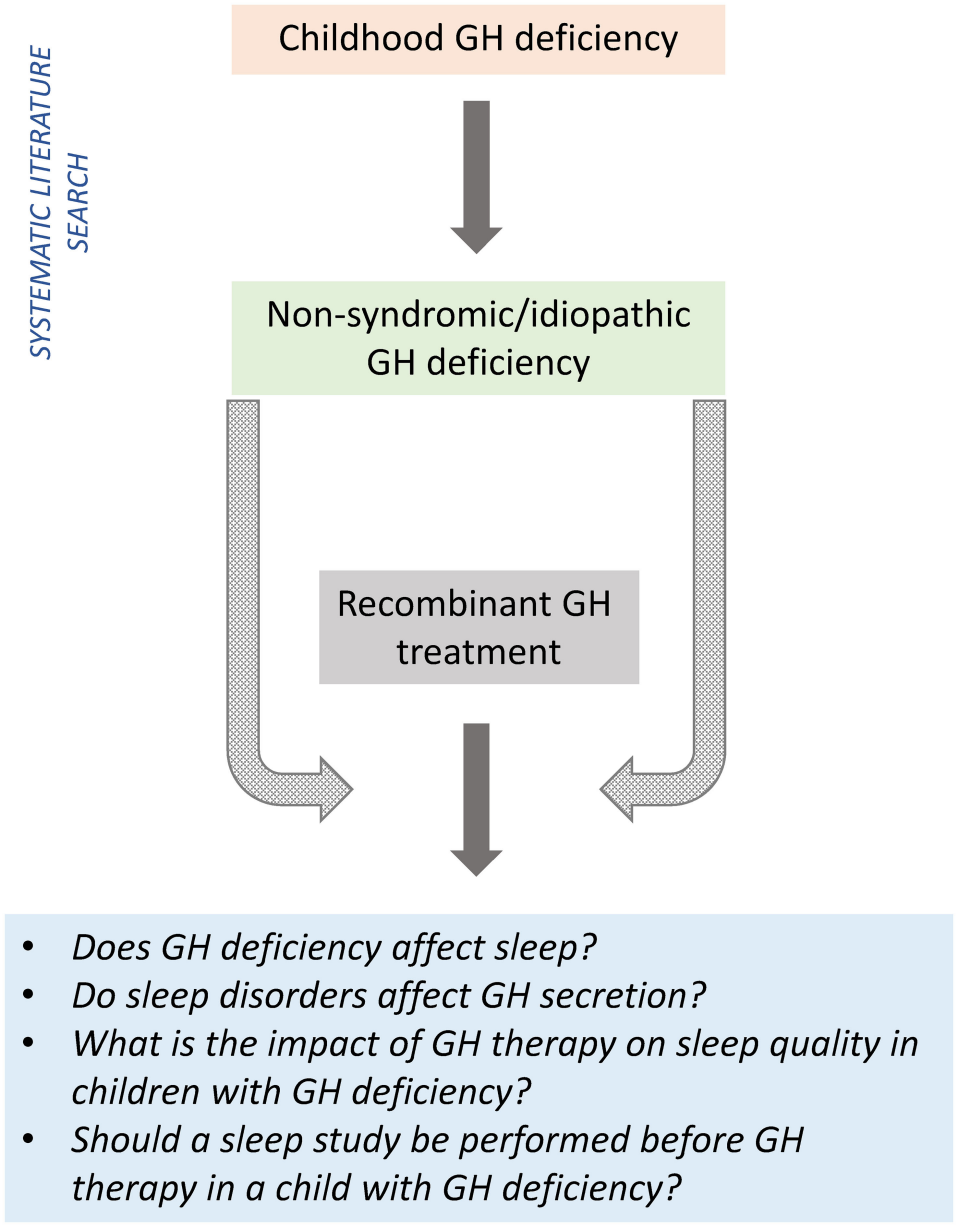
Study rationale and objectives. GH, growth hormone.

This narrative review (incorporating a systematic search of the literature) aimed to assess the relationship between GH and sleep in children, and assess the impact of GH therapy on sleep quality in children with idiopathic GHD (**Figure 1**).

## 2 Methods

### 2.1 Search strategy and data extraction

The Medline PubMed, Scopus, and Web of Science databases were systematically searched for English language articles published up to April 2023. Medical Subject Heading (MeSH) terms and text words (including their combinations and truncated synonyms) were adapted as necessary to search in each database.

In conducting this review, we followed the Preferred Reporting Items for Systematic Reviews and Meta-Analyses (PRISMA) guidelines to ensure rigorous methodology and transparent presentation of data. The phases of study selection, data extraction, and methodological quality assessment were conducted in accordance with the recommendations of the PRISMA protocol.

The exclusion criteria for the literature search were “case report,” “review”, and “syndrome”.

The PubMed search strategy used was: (children[Title/Abstract] OR pediatric[Title/Abstract]) AND (“growth hormone deficiency”[Title/Abstract]) AND sleep[Title/Abstract] NOT (“case report”[Title/Abstract] OR review[Title/Abstract]) NOT syndrome [Title/Abstract]. The Scopus search strategy was ABS (children OR pediatric) AND ABS (“growth hormone” OR “growth hormone deficiency” OR “growth hormone treatment” OR “recombinant growth hormone”) AND ABS (sleep OR “sleep quality” OR “sleep pattern” OR “sleep duration” OR “sleep efficiency” OR “sleep satisfaction”). The Web of Science search strategy was: AB=(children OR pediatric) AND AB=(“growth hormone” OR “growth hormone deficiency” OR “growth hormone treatment” OR “recombinant growth hormone”) AND AB=(“sleep quality” OR “sleep pattern” OR “sleep duration” OR “sleep efficiency” OR “sleep satisfaction” OR “sleep health”) AND AB=(“sleep study” OR questionnaires OR polysomnography) NOT AB=(syndrome) NOT AB=(review OR “case report”).

### 2.2 Data selection, synthesis and analysis

Two reviewers independently extracted data from all eligible studies. Extraction was completed in duplicate to minimize errors and potential biases in result interpretation. Any discrepancies were resolved by a third reviewer, ensuring accuracy and consistency of the data extracted. Reviewers also assessed the methodology of each study, including the robustness of the study design and the validity of the results, to evaluate the overall quality of the scientific evidence. Studies were excluded if they were deemed unrelated to the topics of interest, published in a language other than English, and focused on children with syndromic/genetic disorders.

## 3 Results

**Figure 2:**
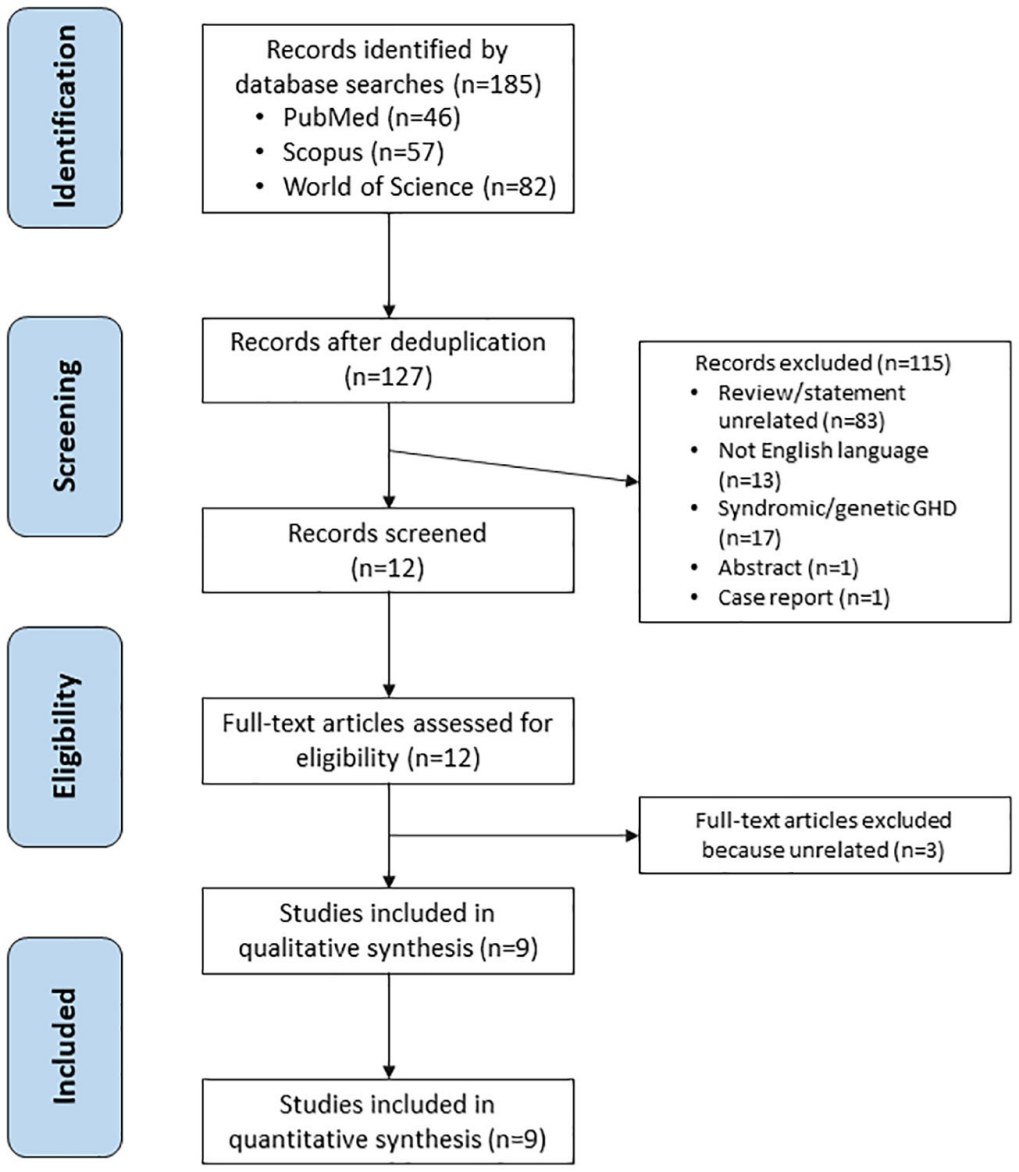
Flow chart showing study selection and inclusion.

The literature search identified 185 articles, of which 58 were duplicates. A total of 127 papers were screened, of which 83 papers were deemed unrelated, 17 focused on children with syndromic/genetic GHD, 13 were non-English, one was an abstract and one was a case report. Twelve remaining articles were assessed for eligibility. After full-text articles assessed for eligibility, three additional studies were excluded (**Table 1**). Finally, nine articles fulfilled the eligibility criteria and were included in the final analysis (**Figure 2**).

**Table 1:** Articles excluded from the analysis after reviewing the eligibility of full-text articles.

Author (Year)	Purpose	Description	Methods	Results
Fagioli, et al. (1991) (14)	To study the effects of radiotherapy on GH secretion and sleep organization in children undergoing cranio-irradiation	Follow-up of 19 children with a history of surgical removal of an intracranial tumor followed by cranial radiotherapy	Simultaneous assessment of SSRGHS, AITT test and polygraphic sleep recording	• Electrophysiological models of sleep phases, quantity, and distribution of sleep phases were normal • In the long-term, SSRGHS and GH responses to AITT were lower after radiotherapy than in the medium-term group • The peak plasma GH concentration corresponded to slow-wave sleep during the first non-REM–REM cycle • There was no correlation between SSRGHS and GH secretion as measured by AITT
Grever, et al. (2000) (15)	To determine the metabolic consequences of GH therapy discontinuation and whether these are related to growth-promoting effects during treatment	Study of 12 children with GHD who had reached their final height *vs* children without GHD; anthropometric and metabolic variables were evaluated after the discontinuation of GH treatment	Assessment of height, weight, skin fold thicknesses, body volume, total body water, basal metabolic rate, and metabolic rate during sleep	• After stopping GH treatment, metabolic effects were observed, including decreased fat-free mass and metabolic rate during sleep • A strong correlation was observed between these and growth-promoting effects during initial GH treatment
Rose, et al. (2001) (16)	To explore factors contributing to sleep disturbance in children with burns	Alterations in circulating neuropeptides, hormones and immunoactive substances known to regulate/modulate sleep were determined, and the effects of drug therapy and interruptions in drug therapy on sleep were evaluated	Review of limited data available, including pathophysiological responses to thermal injury, pain and discomfort experienced, medications used to treat symptoms, and the physical environment in the Burns Intensive Care Unit	• Possible improvement of healing through improved sleep quality, related to GH secretion and tissue repair

AITT, arginine-insulin tolerance test; GH, growth hormone; GHD, growth hormone deficiency; REM, rapid eye movement; rhGH, recombinant growth hormone; SSRGHS, spontaneous secretion of sleep-related growth hormone.

### 3.1 Study characteristics and quality

The nine publications evaluated included six clinical studies, two case series, and one survey; three of the clinical studies included a control group of individuals without GHD and three clinical studies were uncontrolled (**Table 2**). Based on the design of the eligible studies, the methodological quality of the available data was low, and the risk of bias was high. In addition, the studies were old (seven of the nine studies were published before 2000; the most recent was published >10 years ago), and the study populations were highly heterogeneous (both within and between papers). Details of the three potentially eligible articles that were excluded from the final analysis are provided in **Table 1**.

**Table 2:** Summary of studies on sleep-related health outcomes and growth hormone in children with growth hormone deficiency.

Author (Year)	Purpose	Design	Description	Methods	Results
Verrillo, et al. (2011) (17)	To investigate sleep structure in children with GHD and insulin-like GH/growth factor axis dysregulation *vs* normal controls	Clinical study (normal control group)	Study of 10 children with GHD and 20 healthy children, using laboratory PSG recordings	Analysis of sleep architecture parameters and sleep microstructure using CAP	• Children with GHD showed decreased total sleep duration and sleep efficiency, and a global reduction in CAP • Movement time and time in stage 2 non-REM sleep were also reduced *vs* controls
Wu and Thorpy (1988) (18)	To compare sleep EEGs between children with GHD *vs* age-matched controls, and evaluate the effect of GH treatment on sleep EEGs in children with GHD	Clinical study (normal control group)	Study of 7 children with GHD using PSG recordings; data from a historical control group were used for comparison	Comparison of sleep parameters between children with *vs* without GHD, and before and after GH therapy	• Before GH therapy, children with GHD spent more time in non-REM sleep stages 1 and 3, and less time in REM sleep than controls • After GH therapy, there was a significant decrease in stage 3 sleep duration • No other sleep parameters were significantly affected by GH therapy.
Verrillo, et al. (2012) (19)	To compare sleep architecture in children with *vs* without GHD, and evaluate the effects of GH therapy on sleep architecture in children with GHD	Clinical study (normal control group)	Study of 5 children with GHD and 10 healthy children using laboratory PSG recordings	Analysis of sleep architecture parameters and sleep microstructure using CAP	• Children with GHD showed decreased total sleep duration and sleep efficiency, and a global reduction in overall CAP *vs* controls • After GH therapy, there was improved sleep efficiency and increased CAP rate, especially during stage 3 non-REM sleep
Cacciari, et al. (1978) (20)	To evaluate GH secretion during sleep in children with short stature and normal response to drug tests	Clinical study (no control group)	Study of 21 prepubertal children with short stature, with monitoring of GH secretion during sleep after arginine and L-dopa tests	Sleep monitoring by EEG and electrooculogram	• All children showed a GH response of >8 ng/mL in ≥1 of the tests • Secretory episodes with GH >8 ng/mL were observed during sleep in all children • Most secretory peaks occurred during deep sleep (stages 3–4), followed by light (stage 2) and REM sleep • In some cases, no secretory peaks were observed during stages 3–4 sleep, but they were present at other times
Buzi, et al. (1993) (21)	To determine relationships between GH secretion and sleep pattern in children with short stature	Clinical study (no control group)	Study of 18 children with short stature, with monitoring of GH secretion every 15 minutes during sleep, and sleep analysis using EEG	Comparison between GH secretion pattern and EEG-based sleep parameters	• There was no significant correlation between GH secretion parameters and EEG parameters evaluated • Peak GH secretion was not clearly linked to a sleep phase, but was more likely to occur during slow-wave sleep *vs* other sleep stages
Andronikof-Sanglade, et al. (1997) (22)	To identify specific abnormalities in a visuomotor psychological test in children with short stature and GHD	Clinical study (no control group)	Study of 40 prepurbatal children with short stature and without organic diseases using GH stimulation tests, sleep tests and psychological evaluation using the Rey-Osterrieth complex figure	Comparison of endocrinological results, sleep tests and psychological evaluation, and analysis of correlations	• Children with abnormal GH secretion showed abnormalities in the visuomotor psychological test • There was a significant relationship between biological and psychological abnormalities
Gulliford, et al. (1990) (23)	To investigate whether shorted total sleep duration was associated with short stature	Survey	Study of 5145 children aged 5–11 years, using data from a self-administered questionnaire	Total sleep time over a week was estimated using parental responses to the questions about bedtime and wake time for their children	• After accounting for other factors associated with height, there was a weak negative association between sleep duration and height
Guilhaume, et al. (1982) (24)	To determine the relationship between stage 4 and sleep deficit and reversible GHD in psychosocial short stature	Case series (n=4)	Report of 4 children with psychosocial short stature using PSG recordings and hGH secretion	Comparison of sleep recordings before and after the growth recovery period, and assessment of GH secretion using the ornithine test	• Sleep recordings showed a severe deficit of stage 4 sleep and a decrease in the duration of SWS • An improvement in sleep quality was observed after the recovery period, especially an increase in stage 4 sleep • GH secretion was low in the early days of hospitalization but returned to normal during recovery
Hayashi, et al. (1992) (25)	To determine the effects of GHD on sleep	Case series (n=3)	Report of 3 girls with GHD using PSG recording, before and after treatment with hGH	Evaluation of the percentage of time spent in REM sleep and mTMs during sleep	• Before GH treatment, REM sleep proportion and mTMs were abnormal in 2 children • After hGH therapy, there was a slight increase in REM sleep proportion and a normalization of mTMs

CAP, cyclic alternating pattern; EEG, electroencephalogram; GH, growth hormone; GHD, growth hormone deficiency; hGH, human growth hormone; mTMs, submental twitch movements; PSG, polysomnography; REM, rapid eye movement; SWS, slow-wave sleep.

### 3.2 Study data

#### 3.2.1 Studies with a control group

One controlled study comparing polysomnography (PSG) data of children with GHD with age-matched controls found that children with GHD had significantly lower total sleep time, sleep efficiency, movement time, and time spent in rapid eye movement (REM) sleep and stage 2 non-REM sleep, plus a general decrease in electroencephalogram (EEG) arousability (17). In addition, children with GHD had more indicators of sleep fragmentation, including more wakefulness after sleep onset and a higher number of awakenings per hour (17).

In another controlled study, a group of seven children with GHD underwent PSG before and after 1–2 weeks of GH therapy (18). Prior to receiving GH therapy, children with GHD spent more time in stage 1 and stage 3 sleep, and less time in REM sleep, compared with age-matched controls. After initiating GH therapy, children with GHD showed a reduction in the time spent in stage 3 sleep, without any significant change in other sleep parameters (18).

The third controlled study included five children with GHD and 10 healthy controls (19). At baseline, individuals with GHD had a shorter total sleep time and longer wakefulness time than controls (indicating worse sleep efficiency). Children with GHD also spent significantly longer time in stage 1 non-REM sleep and significantly less time in stage 2 non-REM and REM sleep. After ≥6 months of GH therapy there was a significant improvement in sleep latency and sleep efficiency, and a significant decrease in the time spent in stage 3 non-REM sleep. Analysis of sleep microstructure using cyclic alternating pattern (CAP) showed an enhancement of EEG oscillations during the time the children were receiving GH therapy (19).

#### 3.2.2 Studies without a control group

In a study evaluating GH secretion during sleep in children with short stature, peaks of GH secretion were found to occur primarily during slow-wave and deep sleep (20). However, there was no significant correlation between GH peaks during sleep or GH levels during the day and growth velocity (20). Another study of children with short stature failed to identify any significant correlation between GH secretion parameters and EEG parameters (including sleep efficiency and time in different sleep stages) (21). In this study, peak GH secretion was not clearly linked to a sleep phase, but was more likely to occur during slow-wave sleep; the first GH peak was more likely to coincide with wakefulness than with other sleep stages (21). A third uncontrolled study found a significant association between abnormalities in GH secretion (especially low nocturnal GH secretion) and the results of a perceptual motor test (22). The authors concluded that their findings suggested that children with short stature and abnormal GH secretion may exhibit cognitive abnormalities independent of their developmental quotient in visuomotor skills (22).

There were several inconsistencies in the findings of the identified studies. While one study (20) reported that many GH secretory peaks occur during deep sleep (stages 3–4), followed by light sleep (stage 2) and REM sleep, another (21) found no significant correlation between GH peaks and a specific sleep stage. However, a study highlighted a potential relationship between abnormal nocturnal GH secretion and cognitive abnormalities (although this was not the case in all study participants) (22).

#### 3.2.3 Survey

This study was performed as part of the National Study of Health and Growth in 1987, and included 9913 children aged 5–11 years (23). The questionnaire used in the survey was completed by parents and included two questions relating to their child’s sleep duration; data on height standard deviation scores were also obtained. Based on data from 5145 children, a weak but statistically significant association was found between the child’s height and reported sleep duration based on total sleep duration over 1 week (with shorter sleep durations in shorter children) (23).

#### 3.2.4 Case series

Based on PSG data from four children with psychosocial short stature, Guilhaume and colleagues found that these individuals had a gross deficit of stage 4 sleep and a shorter duration of slow-wave sleep than age-matched controls (24). Improvements in sleep quality and an increase in stage 4 sleep duration were seen during a second PSG performed during a growth recovery period (24). In another case series (n=3), PSG was used to evaluated the effect of GHD and its treatment on sleep (25). Before treatment, the proportion of time spent in REM sleep was lower than normal in two of the three children, and was improved after they received treatment with hGH. The same two children also showed a lower than normal number of submental twitch movements during sleep from baseline after starting hGH therapy. The remaining patient showed a reduction in eye movements during REM sleep compared with normal levels, but this was ameliorated by hGH therapy; there was also a tendency for eye movements to increase after hGH administration in the other two patients (25). Furthermore, GH therapy had a beneficial effect on sleep disturbances in some, but not all, individuals with GHD (18, 25).

#### 3.2.5 Excluded studies

The articles listed in **Table 1** did not explicitly investigate the correlation between GH and sleep, nor did they assess the influence of GH therapy on sleep quality. They investigated various topics, such as the effects of radiotherapy on GH secretion and sleep characteristics (14), metabolic consequences during sleep due to interrupting GH therapy (15), and sleep disorders and GH secretion in children with burns (16). These articles do not specifically focus on the relationship between GHD and/or GH therapy and sleep.

## 4 Discussion

The studies identified by a systematic literature search provide interesting insights into the relationship between GH and sleep in children with GHD. They emphasize the importance of monitoring GH secretion during sleep and suggest a link between GHD, sleep patterns, and growth outcomes in specific populations. GHD can impact sleep and lead to cognitive consequences in affected children. Treatment with hGH has the potential to improve sleep disturbances and mitigate adverse effects on cognitive function. However, findings were not always consistent, and the quality of available data was low. Therefore, further research is needed to better understand the effects of GH therapy on sleep in pediatric populations, and the intricate connections between GH, sleep microstructure, and neurocognitive function in this population.

Despite the limitations of the dataset, the available information did provide some insights into the relationship between GH and sleep in children with GHD, allowing us to answer the individual questions listed in **Figure 1**.

GH regulates a variety of processes in the body. Children with GHD show brain structure and function alterations (26). During deep sleep, the body produces and releases the most GH. In children affected by GHD, the production of this hormone may be reduced or absent. Currently available data suggest that this can alter the structure and quality of sleep (18, 25). Even in adults, GHD has been shown to negatively impact REM sleep, body movements during sleep, and sleep microstructure (27). A study conducted in adults with GH disorders showed that all had abnormal REM sleep and delta waves during PSG (28).

According to data from some of the studies identified in the current search, sleep disorders can influence GH secretion in children. The study by Cacciari and colleagues showed that the factors regulating GH secretion during sleep may differ from those that influence GH secretion during wakefulness (20). Buzi and colleagues found that GH secretion during sleep may be associated with specific sleep stages, such as slow-wave sleep, but no significant differences were found in sleep stages before, during, and after the GH peak (21). Another study highlighted the relationship between partial and reversible GHD and sleep disturbances in psychosocial short stature (24). The results of that study suggest that sleep quality improves when growth recovers in a new environment. However, it is important to note that factors other than GH secretion can also influence sleep disturbances in these patients, including age, genetics, nutrition, and overall health status (24). Overall, the mechanisms regulating GH secretion during sleep are not well understood; therefore, there is a need for further evaluation of these relationships, both in children and adults.

It was encouraging to see data showing some sleep improvements when children with GHD are treated with GH replacement therapy (18, 19, 25). GH treatment has been shown to significantly improve sleep quality in adults with GHD (28–30). This can result in deeper and more restful sleep, ultimately improving the individual’s quality of life (30). However, the effects of GH therapy on sleep varied between individuals. While some children may experience significant improvements in sleep, others may only notice small changes or no change at all. It is also worth noting that the effects of GH treatment on sleep may take time to develop. Therefore, it is crucial to have realistic expectations regarding the outcomes of GH treatment on sleep quality. It would also be helpful to know which factors might affect the individual response to GH therapy. Overall, more studies in pediatric patients are needed, including those with a larger sample size conducted in individuals managed according to current guidelines.

Given the association between GHD and sleep disturbances in both adults and children, and acknowledged limitations in the current diagnostic processes (31, 32), a sleep assessment should be included in the evaluation process of children with GHD. The most comprehensive way to achieve this is by using PSG, which provides detailed information about sleep structure, sleep stages, and sleep disorders, such as OSA (33). By analyzing PSG data, researchers and clinicians can better understand the potential impact of GHD on sleep and vice versa. A sleep assessment may also help identify other factors potentially influencing GH secretion (e.g., sleep disorders or altered circadian rhythms) (33). As a result, use of a comprehensive sleep assessment (alongside thorough history taking and laboratory testing) could help inform both a diagnosis and the treatment strategies for children with sleep-related GH disorders or GHD-related sleep disorders.

The key limitation of the current review was the low availability and quality of the published data on the effects of GH on sleep in children. In addition, the lack of consistency in endpoints meant that we were unable to combine and analyze the data in any systematic way. Overall, our report should be considered as a guide to gaps in current literature, and an indication of where future studies are needed.

In conclusion, there is potential for GHD to have a negative impact on sleep in children, including difficulty falling asleep, restless sleep, frequent awakenings, short sleep duration, and poor sleep quality. Sleep disturbances in these individuals can disrupt deep sleep and influence GH secretion. Therefore, it would seem advisable to perform a sleep study (e.g., PSG) before initiating GH therapy in children with GHD. This would enable evaluation of sleep characteristics, identification of any GH-related sleep disorders and development of a personalized management plan. It would also allow changes in sleep during GH treatment to be accurately monitored. This personalized approach will play an important role in improving the overall well-being and outcomes for affected individuals.

## Author contributions

MZ: Conceptualization, Validation, Writing – review & editing. AP: Conceptualization, Validation, Writing – review & editing. PC: Conceptualization, Validation, Writing – original draft, Writing – review & editing. AG: Conceptualization, Validation, Writing – review & editing. FA: Conceptualization, Project administration, Supervision, Validation, Writing – review & editing.
